# Regulation of Food Supplements and Pharmacists’ Responsibility in Professional Practice: A Review

**DOI:** 10.3390/pharmacy14010025

**Published:** 2026-02-03

**Authors:** Cristina Ioana Niculaș, Sonia Bianca Blaj, Marius Călin Cherecheș, Raul Miron, Daniela Cristina Valea, Daniela Lucia Muntean

**Affiliations:** 1 Medicine and Pharmacy Doctoral School, George Emil Palade University of Medicine, Pharmacy, Science and Technology of Targu Mures, 540142 Targu Mures, Romania; cristina.niculas@hotmail.com; 2 Department of Law and Public Administration, George Emil Palade University of Medicine, Pharmacy, Science and Technology of Targu Mures, 540142 Targu Mures, Romania; sonia.blaj@umfst.ro (S.B.B.); raul.miron@umfst.ro (R.M.); daniela.valea@umfst.ro (D.C.V.); 3 Faculty of Pharmacy, George Emil Palade University of Medicine, Pharmacy, Science and Technology of Targu Mures, 540142 Targu Mures, Romania; daniela.muntean@umfst.ro

**Keywords:** food supplement, international regulations, legal responsibility, liability, pharmaceutical malpractice

## Abstract

(1) Background: Regulations governing food supplements vary considerably across countries, allowing products that are prohibited in one jurisdiction to be legally sold in another. Furthermore, online sales enable and facilitate this practice. Regarding pharmaceutical malpractice, the absence of a standardized European framework complicates the evaluation of pharmacist liability. As a result, the specific elements of the liability framework are defined by the national legislation of each Member State. The aim of our review is to map the global regulatory landscape of food supplements and to examine the pharmacist’s professional responsibilities, including instances of malpractice related to this area. (2) Methods: A literature review covering publications from January 2020 to December 2024 was performed using four databases: Scopus, PubMed, Embase, and Web of Science. The search retrieved 8243 records, of which 77 studies fulfilled the eligibility criteria. The extracted data were organized into five main themes: pharmacist responsibility and malpractice, food supplement regulation, consumer safety, health claims, and pharmacist knowledge. (3) Results: The literature reviewed indicated a relatively low number of malpractice cases within the pharmacy profession compared to other professions. A higher incidence of cases is observed among male pharmacists and those practicing in the private sector. Notably, no cases have been identified addressing pharmacists’ responsibilities in the dispensing of food supplements. In the context of food supplement regulation, the reviewed literature highlights a lack of standardized terminology and harmonized legislation across different jurisdictions. Therefore, products may be classified differently across jurisdictions. Another observed barrier is the considerable variation in market access requirements across countries. Regarding consumer safety, several irregularities have been observed. Substantial non-compliance in both product composition and labeling has been observed, reflecting insufficient quality control measures. Concerning health claims, significant regulatory non-compliance with European Union regulations has been documented. In addition, widespread misleading advertising practices have been observed. With respect to pharmacists’ knowledge, the reviewed literature identifies several professional challenges within pharmacy practice, particularly those concerning the dispensing of food supplements. (4) Conclusions: This research offers a comprehensive analysis of the literature published over the past five years concerning pharmaceutical malpractice cases, as well as an examination of food supplement regulation and the professional responsibilities of pharmacists. A recurring barrier identified is the absence of unified regulatory frameworks worldwide. This results in uncertainty concerning the pharmacist’s professional role and responsibilities.

## 1. Introduction

Food supplements are available without significant restrictions in supermarkets, pharmacies or specialized stores. They can be recommended by healthcare professionals but are often self-administered. A common misconception equates natural origins with supplement safety. In reality, risks exist, as evidenced by kava-induced liver damage. Despite being classified as food products, food supplements may exert biological effects and interact with medications. Furthermore, food supplement intake has been associated with adverse reactions, including severe adverse reactions as hepatotoxicity or allergic reactions [1,2,3,4].

Supplement users fall into two main groups: those pursuing prophylaxis against future illness and those addressing existing conditions. Key motivations include enhancing well-being and preventing or managing disease, making supplements more important along with conventional medicines. This has led to fast growth in the markets for herbs, vitamins, and minerals [2].

Despite scant evidence of therapeutic efficacy, over one-third of American and European adults consume daily multivitamin and mineral supplements, predominantly to prevent chronic diseases, including cancer [2]. In Germany, approximately 75% of the population reported using dietary supplements in 2022, with vitamins accounting for 58% of consumption [5]. According to market forecasts, the global food supplements market was estimated at USD 152 billion in 2021 and is projected to reach approximately USD 300 billion by 2028 [6]. Market expansion is driven by population aging and consumer awareness. Population aging increases demand for products supporting bone, joint, and eye health. Consumer awareness is fueled by digital media, including television and websites. They promote preventive health, nutrition and fitness over curative interventions. These trends reflect a steady increase in consumer demand and highlight the growing importance of regulatory oversight and professional responsibility in this sector [2,7].

Although the EU aims to establish uniform regulation, it has not yet achieved consistency across all member states. The Food Supplements Directive 2002/46/EC aims to harmonize standards across Member States by creating a legal and regulatory framework [2]. According to Directive 2002/46/EC, food supplements are defined as “foodstuffs the purpose of which is to supplement the normal diet and which are concentrated sources of nutrients or other substances with a nutritional or physiological effect, alone or in combination, marketed in dose form, namely forms such as capsules, pastilles, tablets, pills and other similar forms, sachets of powder, ampoules of liquids, drop dispensing bottles, and other similar forms of liquids and powders designed to be taken in measured small unit quantities” [8]. These products may contain a wide range of ingredients, including vitamins, minerals, amino acids, essential fatty acids, fiber, plant extracts and other bioactive compounds [8]. According to Article 1 of the Directive 2002/46/EC there is a clear distinction between food supplements (regarded as food products) and medicinal products (defined and regulated by Directive 2001/83/EC). Also, Article 6 of the Directive lays down that for food supplements “The labelling, presentation and advertising must not attribute to food supplements the property of preventing, treating or curing a human disease, or refer to such properties”.

Terminological variations in the classification of food supplements result in divergent regulatory categorizations across jurisdictions. Regulatory ambiguity further complicates the classification of products marketed as food supplements, which may contain naturally occurring active compounds classified also as medicinal products. Certain US supplements are classified as drugs internationally. Global hazards arise from adulteration, contamination, misidentification, mislabeling, or unsubstantiated claims [2,9].

Regulatory requirements for food supplements differ significantly across countries, meaning that a product that may be permitted in one jurisdiction could be subject to stricter conditions or not eligible for sale in another. Since supplements are classified as non-pharmaceutical products, their purchase does not require a prescription. Furthermore, online sales enable broad distribution, including in countries where the products have not been officially registered, thereby circumventing regulatory oversight [10].

In this context, the issue of pharmaceutical malpractice could also arise implicitly, if we can discuss a breach of pharmacists’ responsibilities in these cases. Despite the fact that it has acquired new legal and social connotations, there is no single regulation in this area at European level. Consequently, the liability of pharmacists in terms of the applicable legal basis, the burden of proof, the types of errors that fall within their professional responsibility, and the right to take action to remedy any damage remains governed by the national legislation of each state.

However, there are certain directives and regulations in the European legal order that are relevant in related areas, such as Directive 2011/62/EU [11] of the European Parliament and of the Council of 8 June 2011 amending Directive 2001/83/EC on the Community code relating to medicinal products for human use as regards the prevention of the entry into the legal supply chain of falsified medicinal products, or Commission Delegated Regulation (EU) 2024/1701 of 11 March 2024 amending Regulation (EC) No 1234/2008 [12] as regards the examination of variations to the terms of marketing authorizations granted for medicinal products for human use. However, it can be seen that these legislative acts focus more on the safety of medicines, authorization and marketing authorization, rather than on the responsibility of pharmacists.

Furthermore, it is worth noting that on 17 June 2025, the European Parliament, the Council of the European Union, and the European Commission launched a reform of European pharmaceutical legislation, known as the “Pharma Package”, which aims to revise the existing rules [13].

The aim of this review is to map the global regulatory landscape of food supplements and to examine the pharmacist’s professional responsibilities, including instances of malpractice related to this area.

## 2. Materials and Methods

This review employed the methodological framework for scoping studies proposed by Arksey and O’Malley [14]. A scoping review approach was selected because it facilitates a broad exploration of the topic. Unlike a systematic review, which focuses on a narrowly defined research question and involves rigorous quality assessment of a limited set of studies, a scoping review addresses broader topics by incorporating diverse study designs and evidence types. Its primary purpose is to rapidly map the key concepts underpinning a research area, as well as the main sources and types of evidence available. This review was performed in accordance with the PRISMA-ScR (Preferred Reporting Items for Systematic Reviews and Meta-Analyses) guidelines for Scoping Review. The checklist is available as Supplementary Material.

The methodology followed the five stages outlined by Arksey and O’Malley: Identifying the research question; Identifying relevant studies; Study selection; Charting the data; and Collating, summarizing, and reporting the results.

Stage 1. Identifying the research question

What is the current global regulatory status of food supplements and what are the professional responsibilities of pharmacists, including their roles related to food supplements, adherence to professional standards of practice and the prevention of professional malpractice?

Stage 2. Identifying relevant studies

A review of the scientific literature published between January 2020 and December 2024 was conducted using four databases: Scopus, PubMed, Embase and Web of Science. This five-year period was selected because significant global revisions to food supplement legislation have occurred during this timeframe. Search strategies were developed by two researchers, and the final search was completed on 14 August 2025. The search employed the following keywords: “*dietary supplements*”, “*food supplements*”, “*legal responsibility*”, “*regulation*”, “*pharmacist*”, “*pharmacy*”, “*malpractice*”, “*liability*”, “*negligence*”, and “*misconduct*”. In addition, national online legal databases were reviewed to identify relevant legislative frameworks.

Stage 3. Study selection

Original research articles and review papers published in English were included in this review. Eligible studies focused on the legislative frameworks governing food supplements and on issues related to pharmaceutical malpractice. Publications addressing the professional liability of pharmacists, including those examining their responsibilities concerning food supplements, standards of professional practice and malpractice prevention, were also considered. Furthermore, studies investigating the quality of food supplements and consumer safety were included to provide a comprehensive understanding of the topic.

Publications that did not explicitly address the legislative or regulatory aspects of food supplements were excluded from the review. Studies focusing exclusively on clinical or therapeutic aspects, studies addressing pharmacists’ responsibility and knowledge related to medicine dispensing, as well as those that did not consider the pharmacist’s role, were also excluded. In addition, research relating to other health professions or studies centered on consumer perceptions and knowledge of food supplements were not included in the final analysis.

Following duplicate removal, articles were independently evaluated by two researchers according to inclusion and exclusion criteria. Screening proceeded initially by title and abstract, followed by full-text review. Disagreements were resolved through discussion.

Stage 4. Charting the data

Eligible articles were independently reviewed by two researchers, and the following data were extracted from each study: authors, year of publication, title, journal, DOI, database of origin, topic addressed, study objective and main conclusions. Any disagreements between the reviewers were resolved through team discussions. The extracted data were systematically organized and managed using Microsoft Excel for Microsoft 365 (Microsoft Corp., Redmond, WA, USA)

Stage 5. Collating, summarizing and reporting the results

Eligible articles were categorized into 5 main themes, which were identified and defined collaboratively by the research team. Afterwards, all authors presented the information that corresponded to each thematic area.

## 3. Results

**Figure 1:**
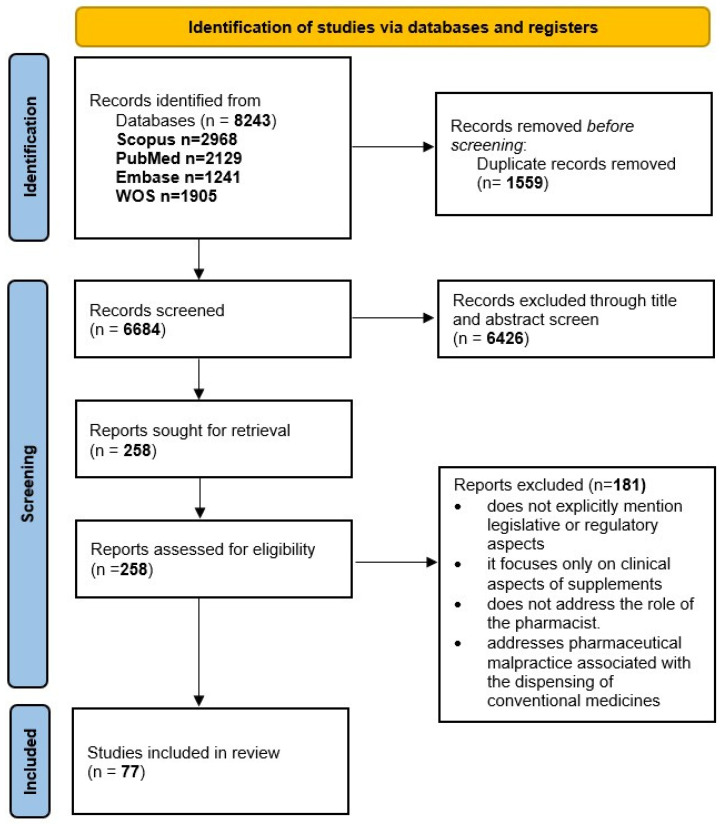
PRISMA flow diagram for academic literature sources.

The systematic search across the four selected databases identified a total of 8243 articles (Scopus *n* = 2968, PubMed *n* = 2129, Embase *n* = 1241, WOS *n* = 1905). Following the identification and removal of 1559 duplicates, 6684 articles remained for analysis. After title and abstract screening, 6426 articles were excluded. At the full-text review stage, 258 articles were evaluated for eligibility and 77 were ultimately selected for inclusion in the final analysis (Figure 1).

Following the literature review, 5 main themes were identified: pharmacist responsibility and malpractice (18 articles), food supplement regulation (26 articles), consumer safety (16 articles), health claims (8 articles) and pharmacist knowledge (11 articles).

### 3.1. Pharmacist Responsibility and Malpractice

#### 3.1.1. Pharmacist Responsibility

With regard to the legal regime and the way in which pharmacist liability is regulated in different jurisdictions, seven articles were identified, namely: pharmacist liability in Romania [15], pharmacist liability in Poland [16,17], two articles on pharmacist liability in Ukraine [18,19] one article on pharmacist liability in South Africa [20], and one on joint physician–pharmacist liability, treated under US law [21].

In general, the violation of legal and professional obligations by pharmacists may entail civil, criminal, or disciplinary liability, as the case may be, but there are certain particularities depending on the specifics of each legislation.

Thus, in Romania, civil liability or malpractice [15] is currently defined in Article 653 (1) (b) of Law 95/2006 [22]. Civil liability does not exclude the criminal liability of the pharmacist if their actions constitute a criminal offense. Furthermore, the regulatory framework does not distinguish between the liability of the doctor and that of the pharmacist, as the conditions for liability are identical from a legal point of view. However, while judicial practice identifies multiple cases of medical malpractice, pharmacist liability is rare. One possible cause is patients’ lack of knowledge, the novelty and diversity of legislation [22,23,24,25], and the difficulty of proving the conditions for liability.

Pharmacy Law No. 266/2008 [23] explains the three forms of pharmacist liability, namely disciplinary, administrative, and civil. However, as we have pointed out, criminal liability cannot be ruled out, as the general conditions prescribed in the Criminal Code are applicable. Liability is proportional to the degree of guilt in the case of co-participation, and the act of the principal is also regulated for the agent. The standard against which the medical act is compared refers to a specialist with basic competence and qualifications, with 10 main responsibilities of pharmacists in pharmaceutical practice in Romania being identified in doctrine: patient counseling, substitution of original medicines with generics, counseling by telephone pharmacy, evaluation of medical prescriptions, collaboration with the doctor to establish therapy; preparation, storage, and administration of medicines; distribution, storage, and supply of medicines; prescribing medicines without the intervention of a doctor in emergencies, clinical pharmacist prescriptions, dispensing medicines in various circumstances, and dispensing OTC medicines; screening and diagnostic services, as well as the administration of vaccines [15].

In comparison, in other jurisdictions, pharmacist liability is subject to more complex regulations. In Poland [16], for example, in matters of professional misconduct, the assessment of pharmacists’ conduct is carried out by the competent authorities of the Pharmaceutical Chamber, with pharmacists being professionally and disciplinarily accountable to the Pharmaceutical Courts, thus establishing a special jurisdiction. Sanctions are imposed by the Supreme Inspectorate of Professional Responsibility and the District Pharmaceutical Tribunal (first instance), and the Supreme Pharmaceutical Court (second instance). The most severe sanction applicable is the deprivation of the right to practice, with the possibility of applying for re-registration on the list of professionally active pharmacists after a period of 10 years. The professional standards identified in domestic legislation are also applicable in Poland. At the same time, the following classification has been identified as an offense involving a qualified pharmacist: The illegal practice of obtaining medicines through a retail route for export is considered a crime and is punishable under the criminal code. Thus, in addition to the penalty imposed by the Pharmaceutical Court, the pharmacist may be imprisoned, which is the most severe penalty at the end of proceedings in the ordinary courts.

In Ukraine, there has been a clear interest in regulating conduct that attracts the criminal liability of pharmacists. In general terms, the following are regulated as crimes infecting a person resulting in incurable infectious diseases, disclosure of employees’ medical information, illegal abortion or sterilization, failure to provide medical assistance, improper performance of professional duties by a medical or pharmaceutical professional, violation of patient rights, illegal experimentation on a person, forced donation, disclosure of professional secrets [18]. The most common offense committed by healthcare professionals in Ukraine is the improper performance of professional duties by a medical or pharmaceutical professional. Thus, in 2018, 99 criminal proceedings were recorded, and in 2019, 130 [18].

The legal framework has several gaps, namely the lack of an objective definition of the concept of medical error, the rights and obligations of medical staff, and the term “improper performance of medical duties”. However, at the time of publication of the article consulted (2020), Ukraine was in the midst of a health reform [18].

In terms of the legal regime, a situation in South Africa that caught our attention is that of the principal’s liability for the acts of the agent in pharmaceutical malpractice. In this regard, according to a study [20], the responsible pharmacist is liable for the actions of the pharmacy assistant, who is also held liable in the event of an error committed by the pharmacy assistant.

Another particular situation is that identified in the United States, where, according to the source consulted [21], the pharmacist’s liability may be engaged in conjunction with that of the physician. This liability derives in particular from the pharmacist’s obligation to inform the patient. The pharmacist has a legal obligation to dispense medicines safely. He or she may be held liable for dispensing a prescription that a pharmacist with basic training would consider unsafe for the patient. Although the doctor is responsible for warning about side effects, the pharmacist must “exercise his own judgment as to whether a prescribed dose, even if confirmed by the prescribing doctor, would be harmful” and has a duty not to dispense a prescription that he considers harmful.

#### 3.1.2. Statistics at the Jurisdiction Level on Cases of Medical and Pharmaceutical Malpractice

The diversity of regulations also shapes judicial practice in this area, in which 11 articles have been identified. However, it is important to note from the outset that a review published in 2024 (2000–2021) [26] concludes that 86% of the studies identified dealing with professional misconduct among healthcare professionals focused on doctors, and of the total studies identified, 60% were conducted in the US and Australia. Only one study [27] was identified that dealt with pharmacists subject to disciplinary measures, which examined the characteristics and risk factors of pharmacists. It follows that the liability of pharmacists is primarily reflected in the disciplinary actions involving them. The lack of public nature of disciplinary procedures can hinder the study of this type of malpractice, posing a systemic risk in this research.

Between 2010 and 2017, a study was conducted in Australia [28] that analyzed decisions regarding serious breaches of professional conduct against healthcare professionals. Another study [29] concluded that at least 6% of registered pharmacists licensed to practice during the study period were the subject of at least one complaint. Of these, less than 1% were the subject of more than 30% of complaints. Over 50% of all complaints resulted in disciplinary measures. Most complaints involved five issues: dispensing of medicines, legality of medicine supply, communication and interpersonal skills, keeping accurate records, and pharmacist health.

In New Zealand [30], the competent authority responsible for resolving complaints against health professionals is the New Zealand’s Health Practitioners Disciplinary Tribunal (HPDT) [31]. With regard to cases of professional misconduct by pharmacists in this country, 58 cases involving 55 pharmacists were identified in the HPDT database between 2004 and 2021.

The most common misconducts were quality and safety issues related to medication, treatment, and care (39.66%). Another problem identified was criminal convictions for criminal activities, found in 31.03% of cases. Of these, 13.79% of cases involved dishonest access/forgery/inappropriate access with the intention of obtaining a financial advantage. As in other jurisdictions, New Zealand also sees a higher incidence of cases involving male pharmacists, which is three times higher than that of female pharmacists [30].

In Canada [32], a study conducted between January 2010 and December 2020 identified 665 cases of malpractice involving pharmacists in nine Canadian provinces. A low rate of disciplinary action was observed, resulting in 1.37 cases per 1000 practitioners per year. The most common cause for disciplinary action was professional misconduct, followed by clinical incompetence and unfair business practices. Ninety-eight percent of cases involved pharmacists practicing in community pharmacies. Improper professional conduct was defined as a violation of standards of practice or legislation governing pharmaceutical practice, but did not include clinical incompetence. Clinical incompetence included any violation related to clinical performance or treatment. Improper/unethical business practices included any violation with financial gain as a motive, such as inappropriate advertising or fraudulent billing. A total of 129 cases were identified that involved single incidents resulting in disciplinary action. Of these, 109 were clinical incidents and predominantly involved dispensing the wrong dose of a medicine or the wrong medicine, as well as the inappropriate dispensing of non-prescription medicines.

In the United Kingdom [33], the competent authority for the disciplinary responsibility of pharmacists is the General Pharmaceutical Council of the United Kingdom. Between 2016 and 2019, 127 cases were published involving investigations due to suspicion regarding fitness to practice, of which 101 involved pharmacists and 26 involved pharmacy assistants. Of the number of practitioners registered during that period, this resulted in a prevalence of 1.8% for pharmacists (out of approximately 55,000 registered) and 1.1% for pharmacy assistants (out of approximately 23,000 registered). Furthermore, the incidence of professional misconduct allegations appears to be higher among male pharmacists than among female pharmacists [34].

In Saudi Arabia [35], the study identified focuses on cases involving complaints of professional misconduct by healthcare professionals that were reviewed by the Medical Professional Misconduct Committee in the Eastern Province of Saudi Arabia between 2016 and 2019. A total of 649 cases of professional misconduct involving 1242 healthcare professionals were identified, of which 69% were found guilty. Of these, 817 (66%) worked in the private sector and 448 (36%) worked in the public sector; 30% were doctors, 53% were men, and 64% were non-Saudi nationals. Of the total number of complaints, 94.5% of those involving pharmacists led to disciplinary action, while 76.6% of those involving dentists and 58.7% of those involving doctors did [36]. There has been an upward trend in complaints regarding disciplinary misconduct in recent years.

### 3.2. Food Supplement Regulation

With regard to the regulation of food supplements, 26 articles were identified that present the regulatory framework at the level of the European Union, the United States, China, Japan, Mexico, Canada, Australia, New Zealand, and the Russian Federation, addressing the following aspects: presentation of the regulatory framework and its evolution, definition and classification of food supplements, authorization procedure, marketing, and liability for adverse effects or injuries. Table 1 summarizes key aspects, synthesized from data extracted from the articles and corroborated with official legislative sources [8,37,38,39,40,41,42,43,44].

A total of four studies [2,4,45,46] present a comparative analysis of the legal provisions in the European Union, the US, and China in terms of the definition of food supplements, their content, and regulatory competence.

With regard to the regulatory system, these studies show that, at the European Union level, a legislative framework has been created through the adoption of EU Food Supplements, Directive 2002/46/EC, and Regulation (EU) No. 231/2012 of the European Commission (e.g., the Directive 2002/46/EC defines supplements as “concentrated products of nutrients or other substances with nutritional/physiological effects, marketed in measured doses (capsules) tablets, liquids” [8]; other aspects such as substances included, specifications for food additives, purity requirements, and information are regulated), Regulations 1169/2011, 1924/2006, and 2015/2283 (labeling and nutritional information). However, the implementation of the Food Supplements Directive is the sole responsibility of the Member States. The legislation of each Member State regulates and enforces aspects related to the list of ingredients, minimum and maximum permitted levels of vitamins, minerals or other substances, placing on the market, permission to enter the territory of a state, the application of penalties for non-compliance, and the confiscation of unauthorized or unregulated substances. However, there is currently no harmonization of legislation among EU Member States. The European Union has not yet established a system of intervention or concrete action, being limited to the obligation assumed by Member States to directly or indirectly apply EU regulations, a European alert system, but one that only allows other Member States to be informed about the entry into the market of an unsafe food product.

The European Union’s regulatory system for botanical products was the subject of a study [47], which showed that herbal products can be marketed in four categories, each with its own legal framework: herbal medicinal products (HMPs) (contain standardized extracts and require marketing authorization, provide comprehensive information on safety, interactions, and contraindications), food supplements (FSs) (are considered food products, are not subject to clear rules on physiological or pharmacological effects, manufacturers are solely responsible for safety, warnings about risks or interactions are usually absent), cosmetics and medical devices (may have medical uses but the main effect must be physiological and not pharmacological, involves different certification systems depending on risk classes).

However, the study notes that despite these regulations, the European Union has so far failed to harmonize regulations on this type of product, which allows the same plants (St. John’s wort, valerian, ginkgo, ginseng, green tea) to be marketed under different categories: plant-derived pharmaceutical products, active pharmaceutical substances, food supplements or cosmetic products, depending on the national regulations of each Member State. Thus, there is a risk that a product containing active pharmacological extracts may be marketed without prior evaluation/authorization. Furthermore, insufficient consumer protection is observed due to the wide variation in the minimum and maximum limits of certain components or dosages, and non-uniform criteria regarding the form of the extract. It should also be noted that there are no interoperable pharmacovigilance databases for supplements at European Union level that would allow the monitoring of adverse reactions and drug interactions. The authors conclude that there is a need for real harmonization and the establishment of common surveillance mechanisms for consumer protection, for the correct and uniform classification of botanical products, and to ensure harmonized and consistent standards for quality and safety [47].

According to the analysis carried out by the authors of a recent study [48], European regulations on supplements for athletes are in line with the provisions of the Codex Alimentarius. A fairly wide variety of nutrients and other ingredients such as vitamins, minerals, amino acids, essential fatty acids, fiber, or other varieties of plants and herbs can be used in the production of sports supplements. Council Regulation 1170/2009 approved “positive” lists for vitamins and minerals. In addition, labels must also include information on how the product contributes to the daily diet.

Even though the use of vitamins and minerals in these products is regulated at European level, the use of other types of substances is left to the discretion of Member States, and upon request, the European Commission may include certain substances on the list of prohibited substances. In practice, there is no uniform regulation at European Union level for substances other than vitamins and minerals.

Also with regard to this issue, in the US, there is federal regulation of dietary supplements under the authority of the FDA and FTC, but approval is not required for their introduction onto the market and marketing. On the other hand, the entire responsibility lies with the manufacturer, who must ensure that the products are safe, correctly labeled, and do not use misleading medical information. The FDA may intervene later if problems arise, provided that it must prove that such a product is unsafe before removing it from the market [45,46].

Furthermore, the DSHEA (1994) defines a dietary supplement as a product intended to supplement the diet and containing vitamins, minerals, herbs, amino acids, or other nutrients [46,49]. Given that it is a continuously growing market (according to study [50], the market has grown from 4000 products in 1994 to 80,000 in 2022), proposals for more rigorous regulation have not been lacking, such as the Supplement Online Wellness Library initiative to establish a voluntary registry for these products [46].

A very recent study (2024) [51] presents the regulatory framework for the labeling of food supplements with reference to ingredients, approaches, and trends in the testing of botanical products marketed in the US to promote immune health, which is the second largest reason for the consumption of these supplements. The study finds that there is growing interest in such products, along with improved consumer behavior, as consumers are becoming increasingly attentive to the authenticity of ingredients and label information. This is a positive development, the study notes, given that popular ingredients (turmeric, elderberry, functional mushrooms) are at high risk of adulteration and mislabeling due to supply chain disruptions during the COVID-19 pandemic.

In the US, the FDA is responsible for post-marketing surveillance and can also verify the compliance and accuracy of labels. These must be truthful and not misleading. Otherwise, the product may be considered mislabeled or adulterated and withdrawn from the market. Food supplements labels must contain five categories of information: identity statement, net quantity statement, nutrition labeling, ingredient list, and name and address of the manufacturer/packer/distributor. The FDA allows six main types of claims, including those related to nutrient content, antioxidants, structure/function (nutritional support) claims, and health claims. The FDA does not allow direct claims regarding immunization or disease prevention but accepts claims such as “supports immune health” provided that the FDA is notified within 30 days of placing the product on the market.

Supplement manufacturers must comply with cGMP standards regarding specifications for each component, including ingredients necessary to ensure specifications for identity, purity, concentration, composition, and contamination limits, as well as existing regulations, which requires at least one test to be performed to establish the identity of ingredients and whether they meet the established specifications. A dietary supplement is considered adulterated if it does not meet the specifications of the finished product, ingredients, or label [51].

Also from a regulatory point of view, China, through the Chinese Food Safety Law enacted in 2015, introduced a new centralized system under the authority of the National Medical Products Administration (NMPA) [9]. This new system permitted the classification of natural products as either medicines, or health foods depending on the product and the claims made; also, regulatory requirements and safety assessment vary. Health foods with health claims need to follow a registration process, while health foods without health claims need to follow a notification process [2,6,9].

With regard to the terminological analysis of the concept of food supplements, a study [9] found that there is a fairly wide range of terminological differences with important consequences for the classification of these products: if a product is considered a food, it cannot have therapeutic indications, whereas if such a product is classified by law as a medicine, it can have therapeutic indications, and an approval procedure is also required and mandatory.

At the same time, several classifications of food supplements have been identified, such as: “food supplements” (EU, Mexico), “dietary supplements” (USA, New Zealand), “health foods” (China), “foods with function claims” (Japan), “natural health products” (NHPs) (Canada), “complementary medicines” (Australia) [9].

For example, green tea, commonly used as an infusion but also in the form of extracts (which actually have a different chemical composition, some of which are associated with liver damage), is classified as a medicine in Canada and Australia, a supplement in Japan, China, New Zealand, and the EU, and as both a botanical medicine and a supplement in the US [9].

Another article [52] also discusses the distinction between functional foods, dietary supplements, and nutraceuticals, drawing a parallel between medicines and dietary supplements and highlighting the existence of important differences between these concepts, which are at high risk of being confused by users. It is important to remember that conventional foods, nutraceuticals and food supplements are used in the prevention of a disease, while medicine is used in the treatment of a disease.

Other aspects regarding the regulation of food supplements were addressed in four studies [53,54,55,56], which considered the labeling of food supplements (with references to their definition), good practice standards, and the marketing authorization procedure.

Thus, in 2004, Germany adopted the German Food Supplement Regulation, which included food supplements in the food category, defining them as foods intended to supplement the diet, with an adequate content and which must comply with the requirements and standards of the European Union in terms of content and quality [53]. In addition, a certain standard of correct labeling is required for marketing, which must include the recommended daily dose, warnings about exceeding it, a warning that the supplement should not replace a balanced diet, the place of origin of the product and the main ingredient, and whether its place of origin is different from that of the finished product. It is important to note the authors’ finding that, in Germany, what qualifies a product as a food supplement is not only the amount of nutrients, but also the presence of ingredients intended to supplement the general diet and the fact that the product is marketed in a dosed form.

A study conducted in Mexico [54] highlights that the country has fairly rigorous regulations on food supplements (General Health Law), which are classified as products based on plants, extracts, and nutrients, but cannot include substances with recognized pharmaceutical or therapeutic properties or plants recognized as toxic. Even if they do not require authorization, they must comply with official standards on good hygiene practices for the processing of food, beverages or food supplements, control of raw materials and warnings on nutritional content, including the statement that “this is not a medicine”.

In comparison, in the US, GMP (Current Good Manufacturing Practice) standards were introduced in 2007, requiring manufacturers to take the necessary measures to prevent adulteration and to verify the identity, concentration, purity, and composition of the supplement in question. Subsequent investigations have shown that not all manufacturers comply with GMP standards. To strengthen control over these products in a rapidly expanding market, the FDA established a new department in 2015—the Office of Dietary Supplements—with oversight responsibilities for dietary supplements [57].

According to the regulatory and supervisory framework outlined by GMP standards, dietary supplements must also contain the following information on their labels: they are not a treatment for any disease and do not prevent the onset of any disease; health claims, which require scientific evidence that the product has an impact on a specific disease/condition and that it is approved or evaluated by the FDA; structure/function claims regarding the influence/effects of the supplement on the body/body function; nutrient content [57].

Alternative programs have been developed and implemented to supervise and monitor the food supplement market, independent of product verification and compliance with manufacturing processes (National Sanitation Foundation International, TRU-ID™ DNA verification) [57].

The same study [54] also analyzes the issue of the authorization procedure for the marketing of food supplements in Canada. These are classified as natural health products (NHPs), which include traditional remedies, food supplements, vitamins, minerals, and herbal products. Unlike other countries on the American continent, marketing requires an authorization issued on the basis of scientific evidence of safety, efficacy, and quality, subject to compliance with good manufacturing practices. In addition to the marketing authorization procedure, the Canadian regulatory system also provides for a post-authorization procedure and consultation with expert committees.

A 2020 study [58] on the regulation of food supplements in Canada highlighted that in 2004 and 2012, more rigorous regulations (considered by manufacturers to be excessive) were adopted regarding product licensing, health claims, the inclusion of warnings and dosage recommendations, annual safety reporting, and the prohibition of the sale of unlicensed products. The study concludes, however, that post-marketing surveillance and adverse reaction management remain insufficient.

Unlike the stricter regulation of food supplements in Canada, in the US the risks to consumers are greater in the absence of a marketing authorization procedure and the obligation to verify efficacy and safety before placing them on the market [56].

Two types of regulations on food supplements have been observed: pre-marketing (relating to safety, efficacy, quality, labeling) and post-marketing (market control and monitoring, reporting of adverse effects, withdrawal of products from the market, sanctions). Each state has imposed its own regulatory system: some are mixed, in which pre- and post-marketing regulations coexist, some focus on pre-marketing regulations, and others focus on post-marketing regulations [59].

Several studies analyzing specific cases regarding the regulation of food supplements are also relevant.

One study [60] analyzed the regulations in 30 countries on food supplements for weight loss, concluding the following: a wide variety of regulations, insufficient control of the marketing of food supplements, which has allowed ineffective, adulterated, or even dangerous products associated with serious adverse reactions to enter the market. However, it was found that certain control mechanisms had been implemented by some states: safety assessment based on scientific criteria; strict labeling requirements; advertising restrictions; reporting of adverse reactions; and product withdrawal from the market.

Three of the studies identified [61,62,63] address the issue of probiotics and their regulation, concluding that there is a wide variety, with large differences between different countries or regions. According to the FAO and WHO, probiotics are defined as “live microorganisms that, when administered in adequate amounts, confer a health benefit” [63]. In some countries, they are regulated as foods, while in others they are regulated as drugs. Very few countries classify them in a separate category and regulate them differently. In general, there is a stricter regulatory framework in Europe, while in the US and Asia the system is more permissive.

Specific to probiotic regulations, beyond classification, there are rules on complete strain characterization, rigorous quality and microbiological risk control, standardized production, complete and accurate labeling, and compliance with rules on monitoring adverse reactions.

A study published in 2023 [64] reveals that supplements for muscle growth (protein, creatine, amino acids) are legal in Canada and widely used, but their regulation has certain shortcomings: there is no minimum age limit for use, insufficient information on risks on the label, and limited control of production, which is only possible after post-marketing reports. However, certain products such as steroids and selective androgen receptor modulators are considered illegal and prohibited. In the US, they are classified as food, and no marketing authorization is required, but in some states, proposals have already been made to ban the sale of these products to minors.

A 2020 study [65] presents the regulation of food supplements in the Russian Federation, where they are classified as food products and are strictly regulated. A mandatory pre-marketing registration procedure is required, even if it does not involve an assessment of the product’s effectiveness. If the product subsequently has health effects that were not considered or declared in the authorization procedure, this is considered false advertising and is subject to liability and penalties.

Federal Law No. 184-FZ also regulates a voluntary certification system to cover such situations, which allows for the inclusion of additional information and provides evidence of product quality and effectiveness following a two-stage procedure: laboratory testing and evaluation of health claims.

Another identified study [66] analyzes how increased financial investment (government, international programs, and collaborations) and human resources (specialization, consultation, communication, and information exchange) can positively influence the development of the food supplement sector and the creation of an appropriate regulatory framework based on improving the standardization of scientific data, evaluation/verification methods, and the harmonization of classification, labeling, and authorization/licensing procedures.

### 3.3. Consumer Safety

With respect to consumer safety, 16 articles were identified, addressing two primary themes: product quality (11 articles) and online marketing (5 articles).

From the product quality perspective, four articles address the topic in general [1,66,67,68], two examine the incidence of adverse reactions [3,69], one focuses on the safety of food supplements intended for athletes [70], one addresses the safety of food supplements aimed at enhancing sexual performance [71], two analyze the quality of food supplements (based on fish oil [7] and resveratrol [72]) and one investigates the authenticity of a food supplement [63].

From the online marketing perspective, one study addresses the marketing of food supplements intended to enhance cognitive function [4], one examines the online marketing of vitamin A-based dietary supplements in the German and United States markets [5], one article discusses misleading information available on the online market in Thailand [73], one study explores the role of social networks in the black market for food supplements [10] and one article addresses illegal online pharmacies [74].

#### 3.3.1. Consumer Safety from the Perspective of Product Quality

In most countries and unlike pharmaceuticals, food supplements generally reach markets without pre-market governmental approval for safety or efficacy. Responsibility for product ingredients and recommended dosages lies with the manufacturer. Driven by regulatory heterogeneity and the absence of systematic oversight, the adulteration of food supplements with pharmaceutical agents represents a critical global issue [5,6]. Two primary forms of adulteration exist: economic adulteration (the incorporation of less expensive substitutes) and pharmaceutical adulteration (the addition of conventional pharmaceutical agents) [1,66].

This phenomenon is perpetuated by the absence of stringent regulations governing food supplements marketing and the ease of online distribution. Between 2010 and 2016, approximately 85% of global food supplements safety alerts were linked to adulteration. In the United States, food supplement use contributes annually to more than 23,000 emergency department visits and 2000 hospitalizations attributable to serious adverse events. A study conducted from 2007 to 2016 in the United States identified 776 adulterated food supplements products, predominantly in categories for weight loss (40.9%) and sexual enhancement (45.5%) [1,71]. Muscle-enhancing supplements were also found to be frequently subject to adulteration. The adulterants include sibutramine, laxatives, amphetamines, and certain antidepressants. The aggressive marketing and consumer belief that supplements prevent chronic diseases drive high rates of usage, despite the lack of proven benefits and the potential for severe side effects, including cardiac issues and liver damage [67].

The presence of adulterated food supplements in the marketplace has been documented, particularly among products designed for exercise enhancement, weight loss and protein powders. This issue is exacerbated by existing regulatory frameworks. Manufacturers are not required to demonstrate product safety prior to market entry, nor that the product contains the composition stated on the label. This regulatory gap permits intentionally or accidentally adulterated food supplements to enter the marketplace [70].

To mitigate the risks associated with non-compliant food supplements, it is recommended to select products certified by an independent testing organization. Such certifications verify the quality and safety of the product, through rigorous evaluation of ingredient content and screening for prohibited substances or pharmaceutical compounds [70].

A 2021 article [71] highlights safety concerns regarding food supplements marketed for sexual performance enhancement. The extensive global consumption of these products is fueled by consumers’ erroneous belief that “natural” formulations are inherently safe. Despite such labeling, adulteration with pharmacological agents is prevalent. Common adulterants include phosphodiesterase type 5 inhibitors (PDE5-i), either alone or in combination, as well as hypoglycemic agents administered at doses exceeding established therapeutic limits. Three distinct cases of hepatic, neurological and cardiovascular toxicity have been reported in association with the use of these products. In France, an ischemic stroke was linked to a supplement adulterated with both sildenafil and tadalafil. The authors conclude that, given inadequate regulatory oversight, these supplements may be classified as counterfeit drugs.

The occurrence of adverse events may result from excessive consumption, allergic reactions to ingredients, interactions with conventional medications and the presence of illicit ingredients and not necessarily from adulteration (Table 2). Regarding toxicity associated with food supplement use, it is primarily attributable to overuse and interactions with conventional pharmaceuticals. Often perceived as a safer alternative to conventional medications, food supplements are frequently consumed in excess, which can lead to undesirable effects, including severe adverse reactions, allergic responses and hepatotoxicity [1,3]. Toxicity examples include nephrotoxicity from Chinese herbal medicines, particularly aristolochic acid, and hyper-vitaminosis D from preparations like Soladek. Additional hazards discussed include risks like fatal cyanide poisoning caused by supplements such as amygdalin [68].

Another contributing factor is the contamination of raw materials or the finished product. The scientific literature highlights the presence of food supplements contaminants such as dust, pollen and toxic heavy metals (e.g., lead and mercury). These contaminants can precipitate severe adverse effects, including poisoning. It is estimated that only 2% of adverse events are reported. Nevertheless, certain adverse events reported by consumers may not result from food supplement use [3,66].

A 2020 study [69] documented severe adverse events associated with food supplement use in the United States. The pharmacovigilance data were obtained from two major manufacturers over 2.5 years. Of 41,121 reported events, 203 were severe, with 69% involved weight-loss products and 19.2% involved glycemic control agents. Symptoms predominantly involved cardiovascular, gastrointestinal and nervous systems.

Severe events were rare (0.48%), with most classified as mild. Concomitant use of multiple food supplements or with conventional medications may contribute to severe outcomes through drug–nutrient or drug–herb interactions affecting absorption, metabolism, or pharmacodynamics. Although clinically significant, such interactions are rarely confirmed with certainty [69].

A 2022 study [3] examined the use of food supplements among US military personnel. The study involved over 26,000 individuals from all military services. Of these, 18% of respondents reported at least one adverse event in the preceding six months. Among those affected, 20% indicated using supplements that contained multiple active ingredients. These products were primarily intended for weight loss, muscle building, and pre- or post-workout enhancement. Heightened risk was associated with female users, smokers, heavy alcohol consumers and individuals with elevated body mass index.

A study published in 2022 [70] examines consumer safety in the sports supplement sector. The study highlights a rising prevalence of product utilization, with the industry marketing more than 50,000 distinct supplements. Nevertheless, it underscores that consumers frequently remain uninformed regarding the potential hazards linked to the consumption of these supplements.

A 2020 study [7] evaluated 10 fish oil-based food supplements marketed in New Zealand. The authors found that 90% of the products complied with existing regulatory standards, yet fewer than one-third delivered all advertised health benefits. All tested supplements were free of mercury. The investigation underscores the difficulties encountered by supplement manufacturers in substantiating health claims. Current legislation permits ambiguous health claim that are challenging to validate and correlate with published clinical evidence.

A study published in 2023 [72] examined 20 resveratrol-based food supplements available on the Slovenian market through pharmacies and specialty stores. The analysis revealed substantial non-compliance in both product composition and labeling. Among the tested supplements, 95% exhibited resveratrol levels different from those declared on the label. Only one product (5%) matched its stated content. Meanwhile, 60% contained higher quantities than indicated, whereas 35% had lower amounts. In addition, 40% exceeded the maximum allowable dose (150 mg/unit, established by the EU for food supplements containing trans-resveratrol, as specified in the Commission Implementing Regulation (EU) 2017/2470 on novel foods), thereby presenting potential health risks. With respect to labeling, although most products included mandatory information, numerous errors and misleading elements were identified. Typographical errors, inaccurate translations, deceptive imagery and unauthorized health claims were identified.

A 2022 article [63] examines the authenticity of the VSL#3^®^ probiotic food supplement. Until 2016, VSL#3^®^ was manufactured according to the clinically validated De Simone Formulation. Thereafter, the distributor introduced an untested alternative formulation while retaining the same brand name. The revised VSL#3^®^ product, manufactured by Actial in Italy, differs entirely from the original version produced in the United States using the De Simone formula. Consequently, the U.S. distributors of VSL#3^®^ were held liable for false advertising. After the US trial, they were found guilty and ordered to pay Professor De Simone over $19 million in damages.

#### 3.3.2. Consumer Safety in the Context of Online Marketing

There is a notable absence of regulatory oversight concerning the online marketing of food supplements. The promotion of a food supplement may be permissible in one jurisdiction yet prohibited in another. Online marketing facilitates the dissemination of such products into countries where the supplement has not undergone required notification [10].

Although online platforms offer convenient access to information, much of it remains unreliable and many supplements contain unauthorized health claims. Recent studies highlight the widespread lack of compliance with current regulations regarding food supplements available online. Significant discrepancies have been observed in regulation, labeling practices, and adherence to recommended daily intake levels [4,5,73,74].

According to an article published in 2024 [4] addressing the online marketing of food supplements, there is an increase in this phenomenon. This form of commerce operates with minimal regulatory constraints, making consumers particularly susceptible to fraud. Online platforms serve dual roles as both shopping tools and informational resources. These platforms additionally enable the spread of false information concerning health and nutrition. Social media cultivates certain psychological states through shared experiences and emotional reinforcement. Research has shown that excessive exposure to content can influence individuals’ emotional states, an effect often amplified by influencers. Moreover, the pursuit of rapid results can promote unethical behaviors. Consequently, the popularity of a product and the perception of control, even when derived from unreliable sources, tend to reduce perceived risk while enhancing perceived benefits [10].

A study published in 2024 [5] offers an example of the practices outline above, as it examined the online marketplace for vitamin A-based supplements available in Germany (75 products) and the United States (26 products). The findings indicated the presence of products containing excessively high doses of vitamin A per tablet, as well as various labeling irregularities.

With respect to labeling, the study identified irregularities in both markets; however, products available in the United States demonstrated greater compliance with regulatory requirements. The identified issues primarily involved inaccuracies in the presentation of the recommended daily allowance (RDA), as well as the absence of information regarding the country of origin or manufacturer. Concerning overdose risk, such potential was observed in 90% of food supplements marketed in Germany and 70% of those available in the United States [5].

A 2024 study examining the online marketplace in Thailand [73] analyzed 332 webpages promoting food supplements. The study found that 85% of these pages contained at least one false claim, while 58% included more than two. The most common irregularities identified were unapproved health claims, exaggerated statements, and inaccurate labeling. Specifically, unapproved claims regarding product benefits appeared in 82.5% of cases, exaggerations related to effects on skin, beauty, or weight loss in 47.9%, and overstatements concerning effects on body structure or function in 42.2%. Additional issues included exaggerated therapeutic or preventive claims, as well as overstatements regarding sexual performance enhancement. The misinformation appeared predominantly in textual form, followed by images and videos.

Regulatory authorities in Thailand face challenges in monitoring and controlling illegal or misleading advertising of food supplements. The high prevalence of misinformation is consistent with global trends concerning false claims about food supplements marketed online. Such practices have the potential to mislead consumers, fostering unrealistic expectations and posing health risks, particularly in cases involving adulterated or illegal food supplements [73].

A study published in 2020 [10] highlighted the role of social media in facilitating the black market for unregistered and illegal food supplements. The findings indicate that Millennials (individuals born between 1981 and 1996) are the most likely to use social media platforms to seek information about food supplements, compare products and make purchasing decisions.

A study published in 2021 [74] underscores the significant risks that illicit online pharmacies pose to consumer safety and highlights the deficiencies in existing regulatory frameworks. These entities are widespread throughout the European Union, dispensing counterfeit products that fail to comply with established standards, smuggled goods and prescription-only medications without appropriate medical authorization.

### 3.4. Health Claims

The search retrieved 8 articles (Table 3). One study focused on the regulation of plant-based food supplements [75], and four studies focused on the analysis of food supplement advertising on Spanish radio [76,77,78,79], revealing significant non-compliance with European Union and Spanish regulations. A similar situation was observed in Poland [80] and the United States [81]. One article addresses the issue of food supplement safety [67].

A study published in 2021 [75] discusses the process of regulating health claims for plant-based food supplements within the European Union. Herbs are currently excluded from the approved list of health claims, pending authorization. Their regulatory classification varies from food supplement to medicinal product, determined by factors such as dosage, formulation and the intended use. The current framework managed by EFSA and the European Commission does not consider the tradition of herbal use. Approximately 80% of products lack approved health claims. This can mislead consumers by fostering a lack of reliable information. The authors propose a graduated approach to scientific evaluation for plant-based supplements. This will ensure safer consumer information, with clinical studies playing a key role in claim assessments.

A 2020 publication [76] presents an analysis of food supplement endorsements aired on Spanish radio broadcasts during the year 2017. In Spain, radio constitutes the most trusted information medium for 82% of the population. A key finding is the heavy reliance on endorsers. Despite legal prohibitions, a notable percentage (40%) of spots use celebrities and experts. The most prevalent endorser was the anonymous spokesperson, followed by celebrities and experts. Explicit claims were more frequent than implicit claims. Rational appeals were used more than emotional appeals. The results indicate that higher credibility endorsers utilize more rational, explicit arguments, while typical consumers favor emotional narratives.

Celebrities or opinion leaders appeared in one in four spots, frequently using testimonial endorsements. They often promote claims related to disease risk reduction. Experts combine product descriptions with recommendations and usage instructions. Doctors are frequently used in radio spots to make specific disease claims, a practice that is generally prohibited under EU legislation. Anonymous spokespeople primarily used product presentations [77,78].

One brand was responsible for the majority of endorsements, representing 80% of the total [77]. Many advertisements promote the use of food supplements over conventional medications, either explicitly or implicitly. They also encourage self-care practices rather than consulting healthcare professionals. Furthermore, consumers frequently inform pharmacists that their purchase was prompted by a specific advertisement [80]. Although scientific evidence on the safety and efficacy of food supplements remains limited, they are frequently portrayed as natural alternatives to the chemical substances contained in conventional medications. A substantial share of the Spanish population believe that food supplements are safe and that the products have undergone evaluation and received authorization from the relevant health authorities. They also hold the belief that food supplements can prevent disease or treat illness in a manner comparable to medicines, while regarding them as more harmless and natural. Their availability in pharmacies leads consumers to assume that these products are both safe and effective [76].

A substantial majority of health claims and mentioned ingredients are unauthorized, often misleading consumers by presenting food supplements as alternatives to medicine. This poses potential health risks due to consumers’ lack of awareness regarding misuse. A total of 80.3% of function claims and 20.4% of disease-related claims were found to be unauthorized under European Union regulations. The research suggests that consumers’ perception of chronic disease is a significant predictor of supplement use as self-medication [79].

Function claims are the most frequent type of advertising message. A substantial prevalence of disease-related claims was identified, appearing in approximately one out of every five advertisements analyzed. Such claims were frequently conveyed in indirect or implicit forms. Additionally, the use of vague claims was observed, many of which can reasonably be characterized as deceptive through omission. These widespread malpractices mislead consumers, leading to incorrect purchase decisions involving products that may be ineffective or potentially harmful when used incorrectly [79].

The research calls for stricter monitoring and penalties to enforce existing regulations and protect the public from deceptive advertising practices [78].

A study published in 2022 [80] examined health-related content in food supplement advertisements aired on 6 Polish TV and radio channels between 9 and 15 March 2020. A total of 46 advertisements were identified, and nearly 30% included unsubstantiated efficacy claims, especially related to weight loss. Although most advertisements complied with legal requirements, 13 (28.2%) featured unreliable assured effectiveness claims. The presence of vitamin D was common, appearing in 17 products. Overall, the results indicate improved compliance with advertising regulations but highlight the continued need for legal reforms to prevent consumer exposure to misleading claims.

A study published in 2021 [81] analyzed marketing claims for 110 weight-loss and muscle-building food supplements purchased from Boston-area retailers in 2013. Key findings reported that most products lacked scientific evidence to support the claims made and often contain ingredients that pose health risks.

On average, products featured 6.5 claims, frequently asserting weight-loss effects despite minimal supporting evidence. Notably, supplements displaying the FDA disclaimer (53.6%) contained significantly more overall claims (7.4 vs. 5.5) and more “results-oriented” claims than those without the disclaimer. Products lacking the disclaimer made more “nutrient claims”. Consumers are largely unaware of the limited regulation surrounding these supplements, as manufacturers are not required to test safety before market entry, contributing to thousands of emergency room visits annually. These results underscore the need for stronger FDA oversight of marketing practices, as many consumers incorrectly assume that such supplements have been pre-approved for safety and efficacy [81].

A study published in 2021 [67] assesses the health effects and risks of selected food supplements. The article underscores that widespread use often lacks strong scientific support for healthy individuals. Key findings show that most vitamin and mineral supplements do not lower cardiovascular disease or cancer risk in healthy people. Omega-3 fatty acids lower triglycerides, but large trials showed no primary prevention benefit for vascular events. Purified EPA showed benefit in secondary prevention for high-risk patients. Weight loss supplements are largely ineffective and potentially dangerous. Chromium showed only minor weight reduction.

### 3.5. Pharmacist Knowledge

Eleven articles were identified that discuss pharmacists’ obligations to provide advice, their professional training, and their specialist knowledge related to recommending or dispensing food supplements. Synthesis of these articles highlights several key themes, including counseling practices for food supplement use, along with pharmacists’ perceptions, knowledge, and challenges associated with dispensing food supplements.

The use of food supplements for disease prevention and management has become increasingly prevalent, positioning these products at the intersection of pharmacy and nutrition. Interest in food supplements has risen considerably, largely due to the perception among consumers that they are safe [82,83].

According to professional standards, pharmacists must respect consumers’ health preferences, support informed decision-making, provide counseling aimed at positive health outcomes, and practice evidence-based care [83]. However, the application of professional standards in pharmacists’ dispensing of food supplements is limited by factors such as time constraints, insufficient resources, and inadequate knowledge [84].

The widespread availability of food supplements in non-pharmacy settings introduces ambiguity regarding pharmacists’ responsibilities for safe use. Additionally, patient counseling is challenged by limited consumer awareness of potential safety risks, as the availability of food supplements in various retail outlets is often perceived by consumers as a guarantee of their safety, reducing the perceived need for pharmacist intervention [84]. Counseling on food supplements should be provided based on the best available evidence when dispensing, subject to consumer consent. If the consumer declines counseling, supplements may still be dispensed, as they are legally considered suitable for self-administration unless, in the pharmacist’s professional judgment, doing so would compromise consumer safety [83].

With respect to pharmacists’ knowledge in dispensing food supplements, a lack of sufficient knowledge among pharmacists was observed. In particular, one study [85] revealed that the Palestinian pharmacists participating in the research demonstrated insufficient knowledge regarding food supplements. Similarly, a 2020 [86] study on weight loss products found that participating pharmacists in Saudi Arabia lacked knowledge of relevant regulations and did not distinguish between products dispensed with or without a doctor’s prescription.

An article published in 2021 [59] addresses the most common problems that lead to insufficient training of pharmacists in the dispensing of food supplements, one of which is the absence of regulations in this area, which leads to uncertainty about their role and responsibility. Other issues identified relate to the discovery of cases of adulteration and incorrect labeling of such products, resulting in reluctance on the part of pharmacists to recommend them, as well as challenges related to the lack of time to provide adequate advice to patients. On the other hand, the same study showed that the obligation of pharmacists to provide advice on the effectiveness of dietary supplements is directly proportional to patients’ expectations of receiving adequate information regarding their safety.

Furthermore, the permissive regulatory framework and lack of standardization in the production and marketing of food supplements can result in variability in their composition and potency, leading to uncertainty about optimal dosing [82]. Plant-based medicinal products produced under regulated conditions benefit from quality control and consistent composition, whereas plant-based food supplements often lack such standards, resulting in variable active ingredients and potential mislabeling [87].

Another identified challenge is the lack of chemical characterization of active compounds in plant-based food supplements. As a result, it is difficult to determine how these compounds may interact with the pharmacokinetics and pharmacodynamics of concurrently administered conventional drugs. Active ingredients in plant-based supplements can have inductive or inhibitory effects on drug metabolism or transport, thereby influencing the absorption, distribution, and elimination of these medications [88,89,90].

Marketing supplements without demonstrating their efficacy or safety, or testing for compatibility and potential interactions of active compounds, often leads to uncertainty regarding the actual content, purity, and effectiveness of these products [87,91]. The scientific literature provides limited evidence regarding the safety and efficacy of active compounds found in herbal food supplements. Information on their mechanisms of action is also scarce, which contributes to healthcare professionals’ limited understanding of potential food supplement–drug interactions [90]. For many food supplements, the absence of robust evidence supporting their effectiveness raises ethical concerns regarding their dispensing by pharmacists [83].

Due to these factors, the use of food supplements presents challenges for healthcare professionals, as these products can affect multiple organ systems and may significantly interact with conventional medications [87]. Additionally, pharmacists frequently approach food supplements with caution since they are typically self-administered and obtained without professional oversight, increasing the risk of potential drug interactions [59].

## 4. Discussion

The literature reviewed [15,16,17,18,19,20,21,26,27,28,29,30,31,32,33,34,35,36] indicates that in most jurisdictions pharmacists may bear civil, criminal or professional liability depending on the nature of the act committed. Responsibility is assigned based on the extent of culpability. Due to the relatively recent adoption of relevant legislation and the inherent challenges in proving the conditions necessary to establish liability, documented cases of malpractice remain infrequent [15,16,17,18,19,20,21,30]. The incidence of reported cases involving pharmacists was lower than that observed in other healthcare professions. A higher frequency of cases has been documented among male pharmacists and those practicing in the private sector [26,27,28,29,30,31,34].

Disciplinary actions most frequently result from violations of the legal and professional standards governing the supply of medicines, unprofessional behavior, and failure to maintain accurate documentation. Additional recurrent grounds for sanctions identified include involvement in deceptive commercial activities and issues related to pharmacists’ own health that may impair practice [30,31,32,36].

In many jurisdictions, professional deontological codes state that pharmacists are fully responsible for the products they supply, including food supplements [15,16,25,84]. The reviewed literature did not reveal any documented cases in which pharmacists were held liable for the dispensing of food supplements.

Within the context of food supplement regulation, the reviewed literature [2,4,6,9,45,46,47,48,49,50,51,52,53,54,55,56,57,58,59,60,61,62,63,64,65,66] highlights a lack of standardized terminology and harmonized legislation across different jurisdictions. Consequently, this disparity has resulted in inconsistent definitions and varied regulatory frameworks worldwide [2,6,9,45,52,53,54,55,56,61,62,63,65,66,83]. Therefore, products may be classified differently across jurisdictions. For instance, within the European Union, the same plant can be categorized as a food supplement, food, medicinal product, or cosmetic [47,55].

An additional problem highlighted is the inconsistent application of Good Manufacturing Practices (GMPs) across different jurisdictions. Nevertheless, the producer is ultimately responsible for the product’s safety, effectiveness, and correct labeling [5,6,51,57,60].

With respect to consumer safety, the widespread perception among the general public is that food supplements are natural products and therefore inherently safe. The reviewed literature [1,3,7,63,66,67,68,69,70,71,72] indicates that, in most jurisdictions, food supplements are marketed without governmental approval for safety or efficacy. This lack of oversight may lead to adulteration with unauthorized pharmaceutical substances, particularly observed in products intended for weight loss, muscle building, and sexual enhancement. It also poses significant health risks, including severe adverse events and potentially harmful drug interactions [1,3,63,66,67,68,69,70,71,72,90,91].

The occurrence of adverse events may result from excessive consumption, allergic reactions to ingredients, interactions with conventional medications, and the presence of illicit ingredients. Another contributing factor is the contamination of raw materials or the finished product with contaminants such as dust, pollen and toxic heavy metals. Additionally, substantial non-compliance in both product composition and labeling has been observed, reflecting insufficient quality control measures [1,3,68].

As indicated by the literature reviewed, limited information is available on the safety, efficacy, and mechanisms of action of the active compounds found in herbal food supplements. With regard to therapeutic effects, the claims made are largely unsubstantiated by scientific evidence. Therefore, determining possible drug–supplement interactions is becoming increasingly difficult for healthcare professionals [6,7,83,88].

With respect to the online market of food supplements [4,5,10,73,74], the reviewed literature highlights widespread lack of compliance with current regulations. The most common irregularities identified were unapproved health claims, exaggerated statements, irregularities in product composition, and inaccurate labeling. In addition, online platforms serve dual roles as both shopping tools and informational resources. The online market provides easy access to information, which is not always accurate or reliable. Also, the legal status of food supplements can vary across jurisdictions, being permitted in some and prohibited in others. A further concern is that online marketing facilitates the introduction of these products into countries without the necessary regulatory approval [4,10,74].

With respect to food supplements health claims, the reviewed literature indicates significant regulatory non-compliance with European Union regulations. In addition, widespread misleading advertising practices have been observed [67,75,76,77,78,79,80,81].

A vast majority of products lack the authorized health claims required under European Union standards. Herbal products are currently excluded from the approved list of health claims, pending authorization. Approximately 80% of products lack approved health claims, resulting in insufficient reliable information and the potential to mislead consumers [75]. Another identified problem is the absence of scientific evidence supporting the claimed efficacy of these products, many of which contain ingredients that may pose health risks. This lack of evidence for efficacy is amplified by significant safety concerns, placing consumers at risk of serious and unverified health effects [67,75].

In the context of food supplement advertising [75,76,77,78,79,80,81], products are frequently promoted through aggressive marketing strategies, including the illegal use of celebrity and expert endorsements. These campaigns often prioritize supplements over conventional medical treatments. This type of advertising, often centered on functional and disease-related claims, creates the impression that these products are effective for nearly any health condition. As a result, consumers may be misled into making incorrect purchasing decisions.

With respect to pharmacists’ knowledge, the reviewed literature identifies several professional challenges within pharmacy practice, particularly those concerning the dispensing of food supplements [59,82,83,84,85,86,87,88,89,90,91].

Various sources emphasize that pharmacists frequently demonstrate reluctance to recommend food supplements. Among the factors identified are their common self-administration and the associated risks arising from inadequate regulatory oversight, potential adulteration, mislabeling, and uncertain purity [59,82]. Another identified problem is the limited scientific evidence supporting the claimed efficacy of these products. Furthermore, as demonstrated in recent studies [92,93], the co-administration of food supplements and medications may affect the pharmacokinetics of certain drugs. There is limited consumer awareness of the potential interaction between food supplements and prescription medications. Moreover, the literature suggests that pharmacists across different settings may possess insufficient knowledge to provide adequate patient counseling [85,86]. However, patients often expect comprehensive guidance on the efficacy and safety of supplements [59].

A strength of this paper is its comprehensive review of the literature published over the past five years (January 2020–December 2024).

A limitation of this review is that the findings obtained from the literature are limited to those available up to January 2025. Additionally, the lack of standardized terminology for food supplements limited the scope of retrieved articles, as the search terms were restricted to “food supplement” and “dietary supplement”. Furthermore, an extensive analysis of the legislation related to pharmacists’ responsibilities and the regulation of food supplements was not undertaken.

### Future Directions

In community pharmacy, food supplements are often dispensed alongside medicines, and patients frequently do not clearly differentiate between these categories regarding safety and effectiveness. Usually, food supplements are chosen or used based on pharmacist advice or supervision, rather than through a doctor’s prescription. Regarding pharmacists’ professional responsibility, many considerations relevant to medicinal products may also apply to the use of food supplements, especially when there are potential interactions or incompatibilities with standard medicines. In this context, additional exploratory studies—both quantitative and qualitative—focused on understanding these mechanisms would be very helpful.

## 5. Conclusions

This research offers a comprehensive analysis of the literature published over the past five years concerning pharmaceutical malpractice cases, as well as an examination of food supplement regulation and the professional responsibilities of pharmacists. A recurring barrier identified is the absence of unified regulatory frameworks worldwide. This results in uncertainty concerning the pharmacist’s professional role and responsibilities.

The results highlight the urgent need for harmonized international legislation addressing both food supplement regulation and the professional responsibilities of pharmacists, including malpractice oversight.

## Figures and Tables

**Table 1:** Food supplement regulations in selected countries.

Country	Name and Classification	Regulatory Agency	Legislation	Pre-Market Approval
European Union	Food supplement, regulated as Food products	EC, National authorities	Directive 2002/46/EC Member States legislation	Notification required, depending on Member State legislation
United States	Dietary supplement, regulated as food products	FDA, FTC	DSHEA (Dietary Supplement Health and Education Act)	Not required, responsibility rests on manufacturer
China	Health food, regulated as special foods	NMPA (National Medical Products Administration)	Chinese Food Safety Law	Notification/Registration (health claims)
Japan	Foods with function claims, regulated as foods	MHLW (Ministry of Health, Labor, and Welfare) CAA (Consumer Affairs Agency) for Supplements	Food Sanitation Act	Notification required, responsibility rests on manufacturer
Mexico	Food supplement, regulated as foods	Ministry of Health The Federal Commission for Protection against health risks (COFEPRIS)	General Health Law	Not required, responsibility rests on manufacturer
Canada	Natural health product, regulated in a separate category	HC (Health Canada), NNHPD (Natural and Non-Prescription Health Products Directorate)	Food and Drugs Act and Regulations Natural Health Products Regulations (NHPR)	Yes, Product License, 8-digit Natural Product Number (NPN) on the label Site License—demonstrate safety and efficacy
Australia	Complementary medicines	TGA (Therapeutic Goods Administration), Territorial regulatory authorities	Therapeutic Goods Act	Notification/Registration
New Zealand	Dietary supplement, regulated as food products	MEDSAFE (New Zealand Medicines and Medical Devices Safety Authority)	Dietary Supplements Regulations	Not required
Russian Federation	Dietary foods/Biologically active food supplements Regulated as food products	Russian Federal Service for Surveillance on Consumer Rights Protection and Human Wellbeing	Russian Federal Law No. 29-FZ on the Quality and Safety of Food	State registration required, responsibility rests on manufacturer

**Table 2:** Determinants of adverse events associated with food supplements administration.

Potential Causes	Product Category	Type of Adulteration/Contamination, Mechanism	Safety Concern
Adulteration (pharmaceutical/economic)	Sexual performance enhancement	PDE5-i, hypoglycemic agents	Hepatic, neurological, cardiovascular toxicity
	Weight loss	Sibutramine, laxatives	Cardiovascular, gastrointestinal symptoms
	Muscle enhancing	Amphetamines, antidepressants	Hepatic, cardiovascular
	VSL#3^®^ probiotic	Economic substitution	Lack of efficacy
Product composition	Fish oil	Discrepancy between claimed health benefits, dosage, and actual efficacy	Lack of efficacy
	Resveratrol	Composition non-compliance (lower amounts than marketed/exceeding maximum allowable dose)	Potential health risks
Contamination of raw materials/finished product	Contaminated products	dust, pollen and toxic heavy metals (e.g., lead and mercury)	Severe adverse effects, including poisoning
Allergic reactions	Products with allergenic ingredients	Presence of allergens in the product	Severe allergic reactions
Interactions with conventional medications	-	May affect absorption, metabolism, or pharmacodynamics	Precipitates adverse reactions
Excessive consumption	Chinese herbal medicines	aristolochic acid	Nephrotoxicity
	Soladek	Elevated concentrations of vitamin D	Vitamin D toxicity
	Products containing amygdalin	Amygdalin	Fatal cyanide poisoning

**Table 3:** Summary of studies retrieved on health claims topic.

Study	Country	Context	Main Issue Identified
Chevallier et al., 2021 [75]	EU	Health claims regulations for plant-based food supplements	Currently excluded from the approved list of health claims, pending authorization
Molina et al., 2020 [76]	Spain	Radio broadcasts (2017)—Analysis of food supplement endorsements	Heavy reliance on endorsers (most prevalent—anonymous spokespeople, followed by celebrities) Explicit claims are more prevalent than implicit claims
Muela-Molina et al., 2020 [77]	Spain	Radio broadcasts (2017)—Analysis of food supplement endorsements	40% of radio spots feature endorsers prohibited by law
Muela-Molina et al., 2021 [78]	Spain	Radio broadcasts (2017)—Analysis of food supplement endorsements	Significant noncompliance with EU regulations
Muela-Molina et al., 2021 [79]	Spain	Radio broadcasts (2017)—Analysis of food supplement endorsements	Extensive use of unauthorized health claims, use of illness as a persuasive strategy, promotion of unsubstantiated benefits
Wierzejska et al., 2022 [80]	Poland	TV and Radio advertising of food supplements (9–15 march 2020)	Nearly 30% of advertised supplements made unsubstantiated effectiveness claims
Hua et al., 2021 [81]	United States	Health claims featured on food supplements promoted for weight loss and muscle building (Boston 2013)	The study identified unsupported health claims
Wierzejska et al., 2021 [67]	Poland, worldwide	Health effects, risks of selected food supplements	Unproven efficacy

## Data Availability

No new data were created or analyzed in this study. Data sharing is not applicable to this article.

## Supplementary Materials

The following supporting information can be downloaded at https://www.mdpi.com/article/10.3390/pharmacy14010025/s1: File S1: Preferred Reporting Items for Systematic reviews and Meta-Analyses extension for Scoping Reviews (PRISMA-ScR) Checklist.

## Abbreviations

The following abbreviations are used in this manuscript:

HPDT	Health Practitioners Disciplinary Tribunal
SAHPRA	South African Health Products Regulatory Authority
US	United States
EU	European Union
FDA	Food and Drug Administration
FTC	Federal Trade Commission
CFSAN	Center for Food Safety and Applied Nutrition
DSHEA	Dietary Supplement Health and Education Act
NMPA	National Medical Products Administration
NHPs	natural health products
GMPs	Good Manufacturing Practices
FAO	Food and Agriculture Organization
WHO	World Health Organization
PFs	probiotic foods
PDSs	probiotic dietary supplements
LBPs	live biotherapeutic products
HMPs	herbal medicinal products
EC	European Commission
EMA	European Medicines Agency
EFSA	European Food Safety Authority
RDA	recommended daily allowance
OTC	over-the-counter medicines
